# The use of balloon‐expandable Sapien‐3 valve in redo aortic valve replacement and the potential risk of left main stem occlusion

**DOI:** 10.1111/jocs.16462

**Published:** 2022-03-31

**Authors:** Thomas Theologou, Sara Clivio, Adel Younes, Stefanos Demertzis, Enrico Ferrari

**Affiliations:** ^1^ Department of Cardiac Surgery Institute Cardiocentro Ticino Lugano Switzerland; ^2^ Faculty of Biomedical Sciences University of the Italian Switzerland (USI) Lugano Switzerland; ^3^ Department of Cardiac Anesthesia Institute Cardiocentro Ticino Lugano Switzerland

**Keywords:** redo aortic valve surgery, Sapien‐3 valve, transcatheter aortic valve

## Abstract

Redo aortic valve surgery for the failure of a previously implanted valve is always challenging. In case of small‐sized implanted valves, the use of a balloon‐expanding Sapien‐3 valve can enhance the final effective orifice area, avoid annulus enlargement complex techniques, and can reduce operative time and morbidities. We describe a case where after explanting a failed 19 mm St. Jude mechanical aortic valve and further deployment of a 23 mm Sapien‐3 valve, the left coronary ostia was obstructed by the skirt of the transcatheter prosthesis. After careful removal of a little part of the skirt, we were able to restore the coronary flow and the patient had a favorable outcome.

## 1 CASE REPORT

**Figure 1:**
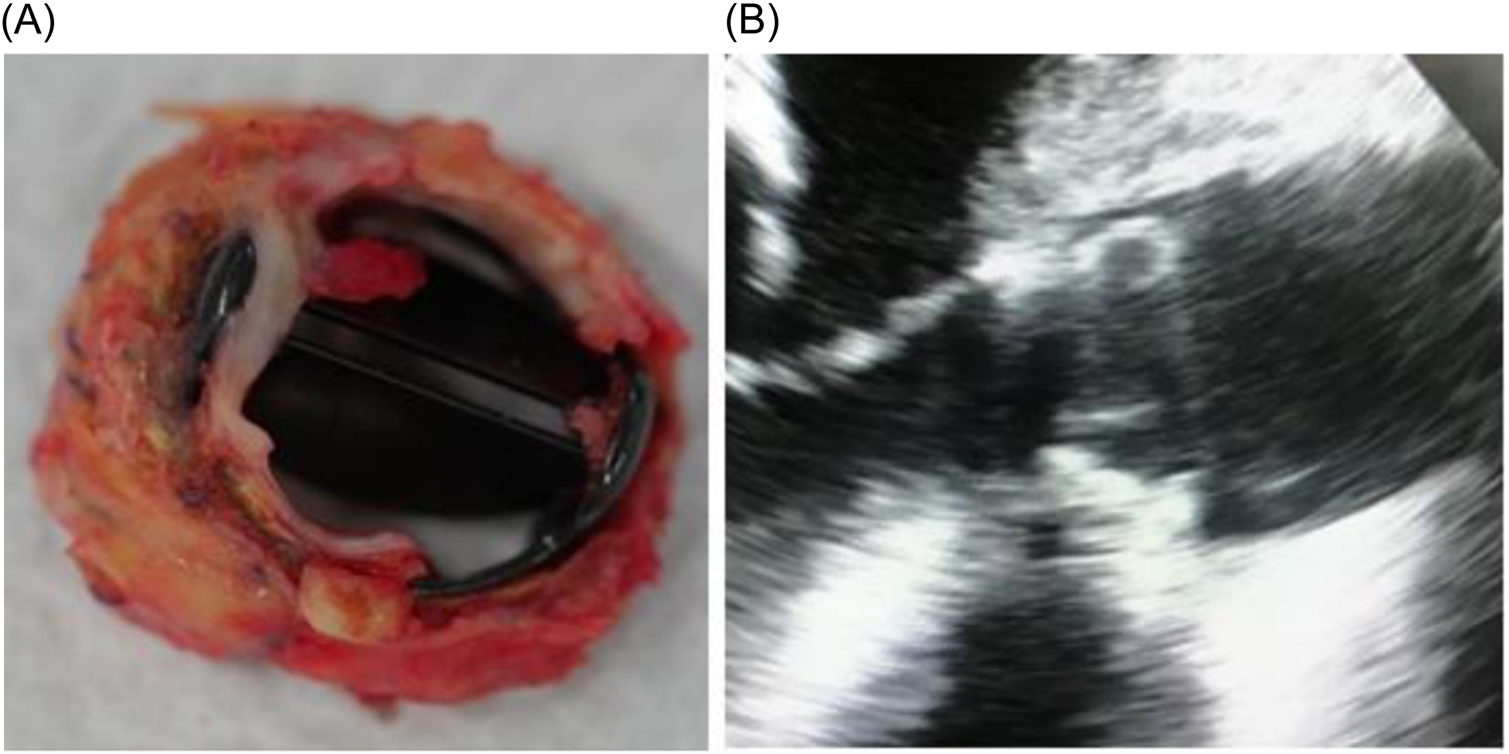
Previous 19 mm St. Jude mechanical aortic valve. (A) Surgical view with pannus and thrombus formation. (B) Echocardiographic view

An 80 years old female admitted to our department for heart failure. In 2011, she underwent an aortic valve replacement with a 19 mm St. Jude mechanical valve (St. Jude Medical, Minneapolis, MN) for severe symptomatic aortic stenosis. A transthoracic echocardiogram and fluoroscopy revealed a thrombus on the mechanical valve with an immobile leaflet, pannus formation, and mean gradient of 45 mmHg (Figure 1A,B).

**Figure 2:**
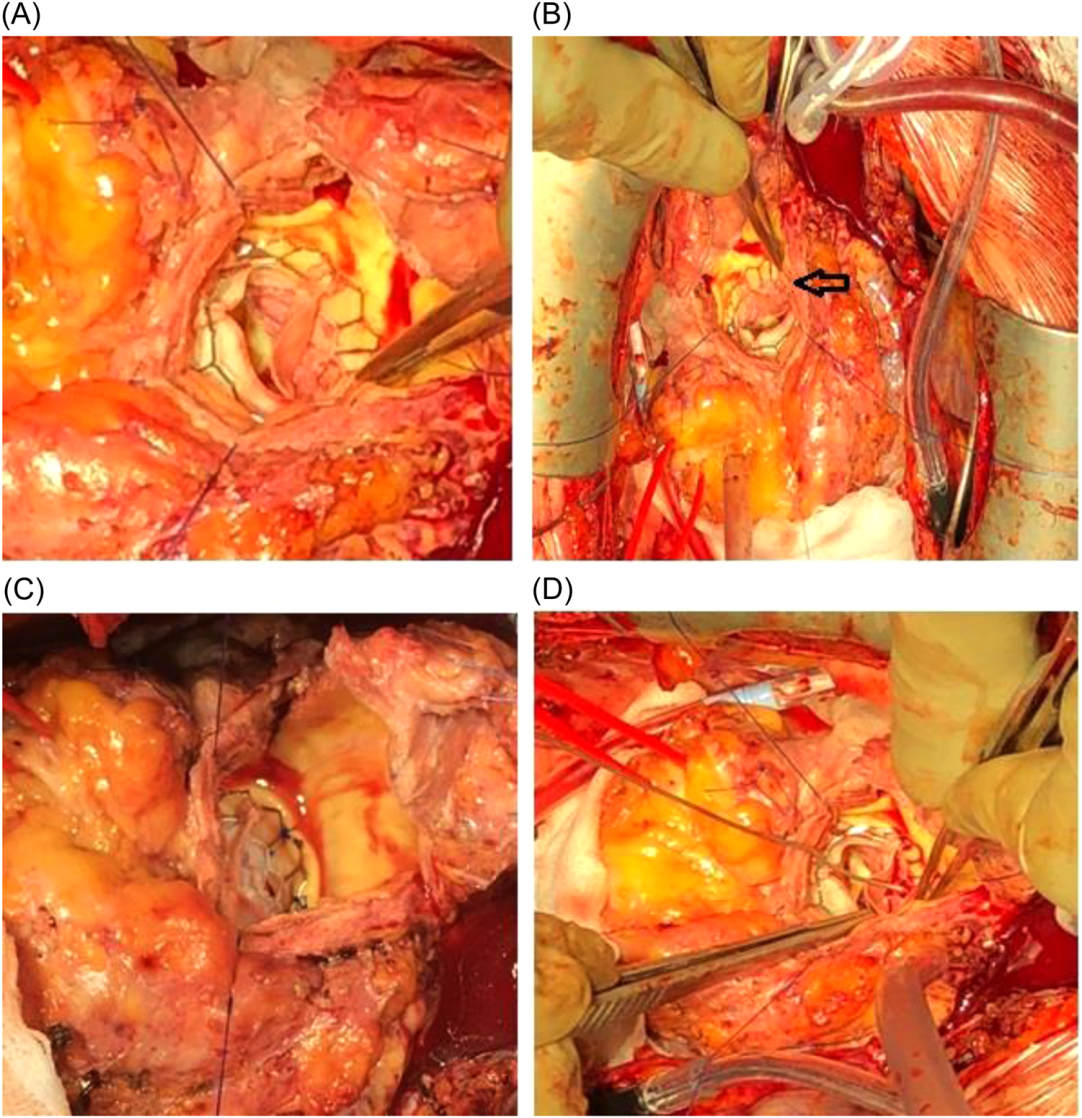
Evidence of obstruction of the left coronary ostia: Clockwise (A and B) (see arrow). Intraoperative field view showing the removal of part of the skirt tissue of the Sapien‐3 valve that was obstructing the left coronary ostia (C and D)

**Figure 3:**
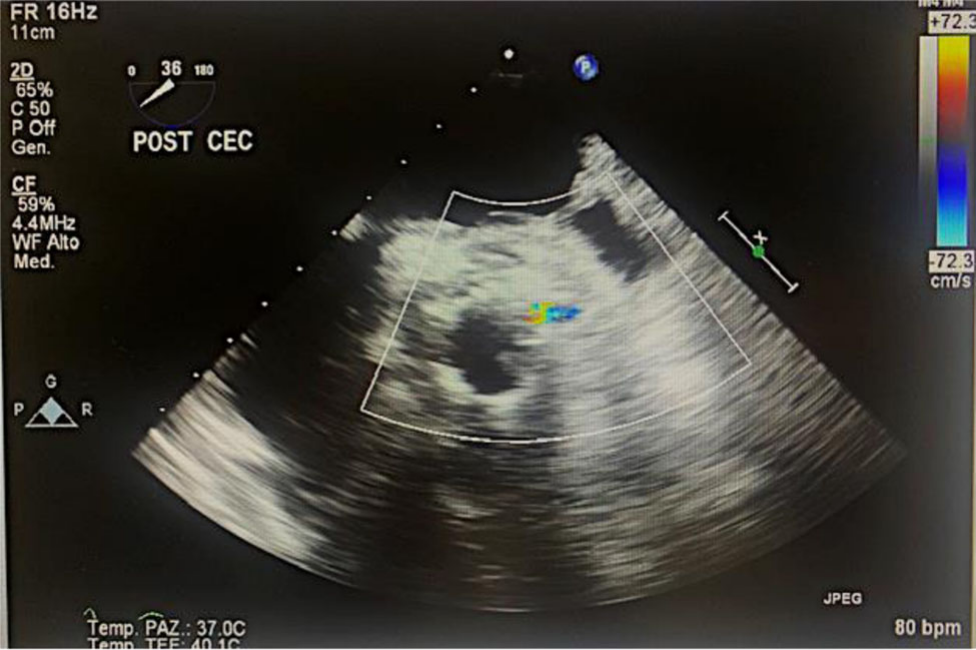
TOE immediate postoperative showing the left coronary ostia free of obstruction with excellent flow

The patient scheduled for urgent redo valve surgery and two coronary bypasses as she presented critical stenosis at the left anterior descending artery (LAD) and the circumflex coronary artery (CX) vessels at the coronary angiogram. The decision of using a transcatheter balloon‐expanding Sapien‐3 valve (Edwards Lifesciences) was determined by the shorter estimated CPB and XC time in this elderly fragile lady (Logistic Euroscore II:42.5%). After completing the bypasses, a 23 mm Sapien‐3 was implanted intra‐annularly under direct vision. However, it was immediately noticed that the skirt of the Sapien‐3 valve was obstructing the left coronary ostia (Figure 2A,B). To avoid this complication, we gently cut a little piece of the skirt of the valve to free the coronary ostia (Figure 2C,D). After successful weaning from cardiopulmonary bypass, the transesophageal echocardiographic examination revealed an excellent flow on the left main stem and normal ventricular function with a mean aortic gradient of 8 mmHg and no AI and no paravalvular leaks (Figure 3). The patient had an optimal recovery and discharged after 11 days with 3 months of Warfarin and aspirin, followed by DAPT for life. Clinical and echocardiographic control after 12, 24, and 36 months showed normal function valve with no AI and paravalvular leaks and excellent flow through the left coronary ostia and improved left ventricular function.

## 2 DISCUSSION

Redo aortic valve surgery in small aortic roots and previously implanted mechanical or tissue bioprosthesis can be a challenge.^1^ The redo operation has three objectives: perform a safe operation with careful access, minimize cardiopulmonary bypass time, and insert a prosthesis with optimal size and gradients to prevent patient prosthesis mismatch.^2^ In the present case, the decision of using a Sapien‐3 valve was based on the increased surface area with low gradients in a small annulus that was accepted already at the previous operation. Moreover, the use of an annulus enlargement technique was not an option as it could be a challenge with increasing surgical times.^3^ An alternative option was to use a sutureless perceval valve. This valve can be used in aortic annuli ranging from 21 to 27 mm diameter, is a self‐expandable bioprosthesis available in four sizes that can be placed in the aortic position during redo aortic valve surgery after the removal of the failed prosthesis. However, the perceval valve has a lower radial force compared to the Sapien‐3 valve and there is a risk, in small annuli, of refolding effect with risk of severe paravalvular leak, not only immediately after surgery but also after some days/months. We, therefore, preferred the Sapien‐3 option in such a small annulus measured 19 mm diameter. In case we would have decided for an annulus enlargement, the sutureless valves are still not recommended.

The last generation of the Sapien‐3 valves instead has proved excellent results, good stability, and easy implantability during valve‐in‐valve procedures. A metanalysis by Tam et al.^4^ suggested that using the valve‐in‐valve technique in high‐risk cases results are comparable with redo high‐risk surgery. Using the expandable valve, studies suggested a less length of hospital stay and less incidence of pacemaker implantation when compared to standard proceduresSimilarly using expandable valves in redo operations prior CABG, the risk of cerebrovascular accidents are shown to be much less in comparison of surgically inserted valves.^5^

Using the transcatheter valve during an open operation, when the failed prosthesis is totally removed leaves a rough area for the implantation of the transcatheter valve. In this contest, coronary obstruction becomes possible, as most of these small roots have been decalcified when the pathological native valve was removed. This can create an abnormal area that can make distortions of the annulus near the coronary ostia. In this case despite the LAD and the CX been bypassed, the proximal septal or lateral territory vessels need some proximal flow from the left main stem vessel to perfuse the first septal and proximal marginal vessels prior to the stenosis or retrogradely from the new grafts. Therefore, careful analysis of the type of valve that needs to be implanted with all the characteristics including the height, size, and structural characteristics is very important. If a coronary obstruction happens with a Sapien‐3 valve, we confirm that part of the skirt can be safely and easily removed to guarantee the flow. Probably, a “surgical model” of the Sapien‐3 valve with no skirt at the level of the coronary ostia can avoid this complication.

## AUTHOR CONTRIBUTIONS

*Concept/design*: Enrico Ferrari and Thomas Theologou. *Data analysis/interpretation*: Adel Younes, Enrico Ferrari, Thomas Theologou, and Stefanos Demertzis. *Drafting article*: Thomas Theologou, Enrico Ferrari, and Sara Clivio. *Critical revision of the article*: Thomas Theologou and Enrico Ferrari. *Approval of article*: Stefanos Demertzis, Enrico Ferrari and Thomas Theologou. *Data collection*: Adel Younes.
